# Population Stratification in the Context of Diverse Epidemiologic Surveys Sans Genome-Wide Data

**DOI:** 10.3389/fgene.2016.00076

**Published:** 2016-05-06

**Authors:** Matthew T. Oetjens, Kristin Brown-Gentry, Robert Goodloe, Holli H. Dilks, Dana C. Crawford

**Affiliations:** ^1^Center for Human Genetics Research Vanderbilt University, NashvilleTN, USA; ^2^Sarah Cannon Research Institute, NashvilleTN, USA; ^3^Department of Epidemiology and Biostatistics, Institute for Computational Biology, Case Western Reserve University, ClevelandOH, USA

**Keywords:** genetic epidemiology, epidemiology, cross-sectional, NHANES, population stratification, EAGLE, global genetic ancestry

## Abstract

Population stratification or confounding by genetic ancestry is a potential cause of false associations in genetic association studies. Estimation of and adjustment for genetic ancestry has become common practice thanks in part to the availability of ancestry informative markers on genome-wide association study (GWAS) arrays. While array data is now widespread, these data are not ubiquitous as several large epidemiologic and clinic-based studies lack genome-wide data. One such large epidemiologic-based study lacking genome-wide data accessible to investigators is the National Health and Nutrition Examination Surveys (NHANES), population-based cross-sectional surveys of Americans linked to demographic, health, and lifestyle data conducted by the Centers for Disease Control and Prevention. DNA samples (*n* = 14,998) were extracted from biospecimens from consented NHANES participants between 1991–1994 (NHANES III, phase 2) and 1999–2002 and represent three major self-identified racial/ethnic groups: non-Hispanic whites (*n* = 6,634), non-Hispanic blacks (*n* = 3,458), and Mexican Americans (*n* = 3,950). We as the Epidemiologic Architecture for Genes Linked to Environment study genotyped candidate gene and GWAS-identified index variants in NHANES as part of the larger Population Architecture using Genomics and Epidemiology I study for collaborative genetic association studies. To enable basic quality control such as estimation of genetic ancestry to control for population stratification in NHANES san genome-wide data, we outline here strategies that use limited genetic data to identify the markers optimal for characterizing genetic ancestry. From among 411 and 295 autosomal SNPs available in NHANES III and NHANES 1999–2002, we demonstrate that markers with ancestry information can be identified to estimate global ancestry. Despite limited resolution, global genetic ancestry is highly correlated with self-identified race for the majority of participants, although less so for ethnicity. Overall, the strategies outlined here for a large epidemiologic study can be applied to other datasets accessible for genotype–phenotype studies but are sans genome-wide data.

## Introduction

In case-control studies, spurious associations can occur when genetic ancestry is associated with both the disease as well as the genetic marker (Cardon and Palmer, 2003). A classic example of a spurious association resulting from population stratification is the association of haplotype Gm and type 2 diabetes among Native American cases and controls (Knowler et al., 1988). Upon closer examination, the cases and controls were not matched by genetic ancestry (proportion of European and Native American admixture) and the frequency of the Gm haplotype differed dramatically between Native American and European-descent populations (Pritchard et al., 2000; Cardon and Palmer, 2003). Both type 2 diabetes and the frequency of the Gm haplotype were associated with Native American ancestry, and failure to account for the ancestry differences resulted in a false positive association with disease status.

Population stratification can be addressed at both the study design and data analysis levels. When designing a genetic association study, investigators should take care to collect at least self-identified race/ethnicity data (Liu et al., 2006), which is highly correlated with broad range genetic ancestry for many groups such as European Americans and African Americans (Tang et al., 2005; Banda et al., 2015). With these basic data, cases and controls can be matched and stratified by race/ethnicity for subsequent tests of association. In lieu of and/or in addition to self-reported race/ethnicity, investigators can use genetic marker data such as ancestry informative markers (AIMs) to estimate genetic ancestry. A major advantage of genetic data over self-reported race/ethnicity is that the former can be used to stratify data like self-identified race/ethnicity but can also be used to adjust statistical models of association between alleles and phenotype and/or identifying outliers (Falush et al., 2003; Price et al., 2006). Indeed, estimating genetic ancestry has become a standard step in quality control in large genome-wide association studies (GWAS) where a hundreds of thousands to millions of genetic markers are available (Turner et al., 2011).

Despite the seeming ubiquity of GWAS data, there are many large DNA collections that lack these dense genetic data for basic quality control and adjustments for population stratification necessary for genetic association studies. One such epidemiologic collection is the National Health and Nutrition Examination Surveys (NHANES) conducted by the National Center for Health Statistics (NCHS), Centers for Disease Control and Prevention (CDC; Chang et al., 2009). NHANES is a cross-sectional national survey that is representative of the US civilian non-institutionalized population. NHANES has collected a wealth of individual sociodemographic and clinical data that has been amassed into a generalizable account of the health and nutritional status of the US. Many of the common chronic diseases with devastating consequences on the US population have been shown to have high heritability indices, indicating a genetic component. To facilitate the investigation of the genetic factors behind these traits, CDC extracted DNA from biospecimens collected as part of the surveys conducted between 1991–1994 and 1999–2002 (*n* = 14,998) and allowed for candidate gene analyses with limited genotyping (McQuillan et al., 2003). Extensive genotyping, including GWAS-level genotyping, has not been permitted on NHANES DNA samples outside of a CDC contract due to participant confidentiality concerns (Centers for Disease Control and Prevention, 2010).

We as the Epidemiologic Architecture for Genes Linked to Environment (EAGLE) study, a study site of the Population Architecture using Genomics and Epidemiology (PAGE) I study (Matise et al., 2011) accessed NHANES for limited genotyping of GWAS-identified variants for replication, generalization, and gene-environment studies. In the absence of GWAS-level data, we describe here the content and usefulness of candidate gene-level data available in NHANES for estimating quality control metrics necessary to avoid population stratification in genetic association studies. The strategies outlined here may be useful to other large-scale epidemiologic or clinic-based collections with limited genetic data available.

## Materials and Methods

### Study Population

For the PAGE I study, EAGLE accessed the Third NHANES (NHANES III) in which DNA was collected in phase 2 (1991–1994, *n* = 7,159) and NHANES 1999–2002 (*n* = 7,839). EAGLE also accessed NHANES 2007–2008 (*n* = 4,611) but only one nuclear marker has been genotyped in this dataset (Crawford et al., 2014); thus, this dataset was not included in the present study. NHANES III collected DNA on participants 12 years and older while NHANES 1999–2002 collected DNA on participants 20 years and older. All study procedures were approved by the CDC’s Ethics Review Board and written informed consent was obtained from all study participants. The NHANES data were accessed for study without identifiers and therefore were considered non-human subjects research by the Vanderbilt University Institutional Review Board.

National Health and Nutrition Examination Surveys collects self-described race and ethnicity on participants. In NHANES III, participants self-identified as “white,” “black,” or “other” for race and “Mexican American” or “not Hispanic” for ethnicity. The variables were combined by CDC for a single race/ethnicity variable: non-Hispanic white, non-Hispanic black, Mexican American, and other. In NHANES 1999–2002, the definition of Hispanic was expanded to include participants who self-identified as Hispanic but not Mexican American (“other Hispanic”). The analysis described here is limited to participants of three self-reported races/ethnicities: non-Hispanic white (NHW), non-Hispanic black (NHB), and Mexican American (MEX). The sample sizes for these three race/ethnicities were 2,630 (NHW), 2,108 (NHB), and 2,073 (MEX) for NHANES III and 4,003 (NHW), 1,350 (NHB), and 1,877 (MEX) for NHANES 1999–2002. And additional 348 (NHANES III) and 609 (NHANES 1999–2002) samples were available from participants who self-identified as “other” and “other Hispanic” but not included in this present study.

### Genotyping

We accessed 411 and 295 autosomal SNPs genotyped in NHANES III and NHANES 1999–2002 study populations, respectively, after linkage disequilibrium pruning (**Supplementary Table S1**). The EAGLE study either accessed existing genetic data in NHANES (Chu et al., 2009; Keebler et al., 2009) or genotyped NHANES DNA samples directly using a variety of assays including TaqMan, Open Array, Sequenom iPLEX^®^ Gold MassArray, and Illumina GoldenGate available in the Center for Human Genetics Research Open Wet Lab and DNA Resources Core at Vanderbilt University. SNPs were selected as candidate gene tagSNPs (Crawford et al., 2006, 2010, 2015; Limdi et al., 2010; Dumitrescu et al., 2011b; Jeff et al., 2012, 2015) or GWAS-index variants reported in the literature and the NHGRI GWAS catalog (Welter et al., 2014) for a variety of common human diseases and traits including but not limited to cardiovascular disease, lipid and inflammation traits, type 2 diabetes, age-related macular degeneration, obesity and body mass index, and bone mineral density and osteoporosis (Matise et al., 2011; Crawford et al., in preparation). All genetic variants available in NHANES including those described here can be found on the CDC website^1^^,^^2^ .

All data were subjected to basic quality control metrics including concordance of blinded duplicates supplied by CDC and checks of Hardy Weinberg Equilibrium stratified by self-reported race/ethnicity. All SNPs included in this analysis were in Hardy–Weinberg Equilibrium (*p* > 0.001) in at least one NHANES subpopulation, and genotype call rates were at least 90%. We pruned the data so that the pairwise *r^2^* of these markers across all comparisons within a 250 kb window was equal or less than 0.80. More stringent *r*^2^ thresholds of 0.50 and 0.10 yielded 361 and 266 SNPs in NHANES III, respectively, and 269 and 226 in NHANES 1999–2002, respectively, underscoring the overall SNP selection process of the EAGLE study.

### Statistical Methods

Minor allele frequencies (MAFs) were calculated stratified by self-identified race/ethnicity and were binned into three groups: monomorphic and rare (<5% MAF), MAF 5–25%, and MAF > 25%. MAF distributions were then compared between NHANES III and NHANES 1999–2002 for each self-identified race/ethnicity. Distributions were also compared across the three major racial/ethnic groups within each survey. Statistically significant differences in MAF were identified using chi-square tests (2x3 and 3x3 tables). Population differentiation was measured by the fixation index (FST), which was calculated using the formula developed by Weir and Cockerham (Holsinger and Weir, 2009).

Processing of genotype files into the input format for STRUCTURE and principal component analysis (PCA) analysis was performed with the genetic analysis, translation, and organization software PLATO (Grady et al., 2010). To estimate genetic ancestry, we applied STRUCTURE (Version 2.3.4) to cluster participants into discrete populations (Pritchard et al., 2000; Falush et al., 2003, 2007; Hubisz et al., 2009). For references of continental ancestry, we downloaded HapMap 3 individual-level genotype data and merged the genotypes with the NHANES dataset. HapMap 3 is the third phase of the International HapMap project and consists of 1,301 samples from 11 populations (International HapMap 3 Consortium, 2010). For this study, we downloaded data for CEU (Utah residents with Northern and Western European ancestry from the CEPH collection), YRI (Yoruba in Ibadan, Nigeria), CHB (Han Chinese in Beijing, China), and JPT (Japanese in Tokyo, Japan). These HapMap 3 samples represent the official reference samples used for genotyping controls in the PAGE I study (Matise et al., 2011). SNPs that did not overlap between the NHANES and HapMap 3 samples were excluded from the analysis. We set the population count parameter *K* = 3 and used the population info flag for only the unrelated HapMap 3 samples [112 CEU, 113 YRI, 170 JPT + CHB (ASN)] representing the continental ancestries.

By setting the migration prior parameter to near zero (10^-10^), we anchored HapMap 3 samples into discrete clusters and allowed the NHANES samples to partition themselves iteratively. Each dataset was run with a round of 10,000 burn cycles followed by 50,000 reps. Eigenvectors were generated by the princomp function and plotted with the GGPlot2 package (Wickham, 2009) in R 2.15.1 (R Core Team, 2013).

## Results

### Genetic Markers Available in NHANES

A total of 411 and 295 autosomal SNPs were accessed in NHANES III and NHANES 1999–2002, respectively. As described in Methods, SNPs selected for genotyping represent primarily GWAS-identified variants for a variety of common human disease and traits as of 2009 (Matise et al., 2011). A greater number of SNPs are available in NHANES III as this dataset was also accessed for earlier candidate gene and GWAS replication studies for lipid and inflammation traits (Crawford et al., 2006, 2010, 2015; Limdi et al., 2010; Dumitrescu et al., 2011a; Jeff et al., 2012, 2015).

For both NHANES III and 1999–2002, the majority of markers available in any self-identified group are common (MAF 5% or greater; **Table 1**). The distribution of minor MAFs, however, differed significantly between NHANES for both non-Hispanic whites (χ^2^ = 14.14; df = 2; *p* = 0.0009) and Mexican Americans (χ^2^ = 12.85; df = 2; *p* = 0.0016) owing to fewer rare and monomorphic variants available in NHANES 1999–2002 (3 and 4%, respectively) compared with NHANES III (10 and 11%, respectively; **Table 1**). Likewise, the distribution of MAFs differed significantly across the three self-identified racial/ethnic groups for both NHANES III (χ^2^ = 17.01; df = 4; *p* = 0.0019) and NHANES 1999–2002 (χ^2^ = 15.26; df = 4; *p* = 0.0042).

**Table 1:** National Health and Nutrition Examination Surveys (NHANES) sample sizes of self-reported racial/ethnic groups and marker distribution by minor allele frequency.

	NHANES III	NHANES 1999–2002
Non-Hispanic whites (NHW)	2,622	4,003
Monomorphic	6%	1%
MAF < 5%	4%	2%
MAF 5–25%	46%	46%
MAF > 25%	43%	51%
Non-Hispanic blacks (NHB)	2,105	1,350
Monomorphic	1%	0.3%
MAF < 5%	7%	7%
MAF 5–25%	51%	52%
MAF > 25%	41%	41%
Mexican Americans (MEX)	2,067	1,877
Monomorphic	5%	1%
MAF < 5%	6%	3%
MAF 5–25%	37%	43%
MAF > 25%	52%	53%


*NHANES III includes 7,159 DNA samples, of which 2,630; 2,108; 2,073; and 348 are from participants that self-identify as non-Hispanic white (NHW), non-Hispanic black (NHB), Mexican American (MEX), or other. Seventeen NHANES III samples (eight, three, and six among NHW, NHB, and MEX) failed genotyping. NHANES 1999–2002 includes 7,839 DNA samples, of which 4,003; 1,350; 1,877; and 609 are from participants that self-identify as non-Hispanic white (NHW), non-Hispanic black (NHB), Mexican American (MEX), or other/other Hispanic. After quality control, a total of 411 and 295 nuclear markers were available in NHANES III and NHANES 1999–2002, respectively. MAF, minor allele frequency.*

### Identification of Ancestry Informative Markers

**FIGURE 1:**
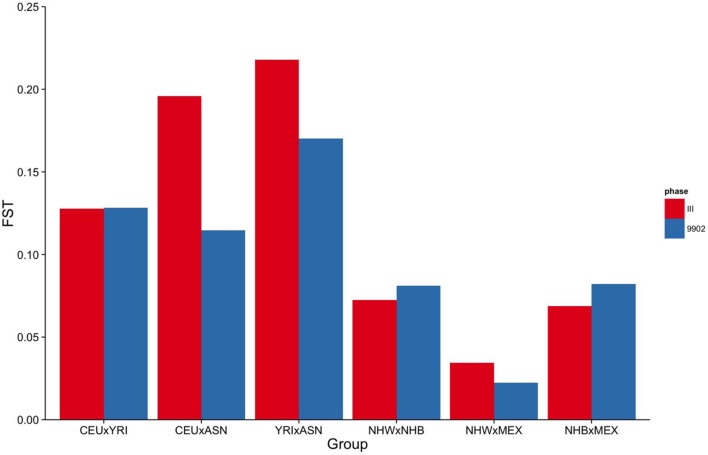
**Fixation Index (FST) of markers genotyped in NHANES III and NHANES 1999–2002.** Mean pairwise FSTs between HapMap 3 populations for markers genotyped in NHANES III (red) and NHANES 1999–2002 (blue).

To measure how informative the markers available in NHANES III and 1999–2002 are for estimating genetic ancestry, we calculated pairwise fixation index (FST) between racial/ethnic groups in HapMap 3, NHANES III, and NHANES 1999–2002 (HapMap 3: CEU × YRI; CEU × ASN; YRI × ASN; and NHANES III and 1999–2002: NHW × NHB; NHW × MEX; NHB × MEX). FST is a measure of population differentiation based on the variance of allele frequencies in a subpopulation compared with the total population (Holsinger and Weir, 2009). As expected, the mean pairwise FST between HapMap 3 groups (**Figure 1**) revealed that a portion of the observed marker allelic variability is explained by population structure.

To estimate the effectiveness and equivalence of our AIMs in the two surveys to estimate ancestry, we compared the FST values measured across HapMap populations. The mean pairwise FST between CEU and YRI was consistently 0.12 when using either the NHANES III or the NHANES 1999–2002 markers, suggesting they are both equally effective at measuring the population structure across these two groups (**Figure 1**). In contrast, the ASN HapMap population consistently presented higher FST values in NHANES III than 1999–2002 with the CEU population (0.20 vs. 0.11) and the YRI population (0.22 vs. 0.17). The lower FST detected in the CEU-ASN and YRI-ASN comparisons by the NHANES 1999–2002 markers suggests that our estimates of admixture and population structure are likely to be underestimates. We also note significantly lower mean FSTs between African and European descent ancestral groups in NHANES (NHW × NHB) compared with the HapMap 3 populations (CEU × YRI; NHANES III: *p* = 2.76 × 10^-10^, NHANES 1999–2002: *p* = 1.27 × 10^-6^). This latter finding reflects European admixture found in African American populations resulting in a reduction of allelic diversity between the two groups.

### Estimates of Global Admixture and Concordance with Self-Identified Race/Ethnicity in NHANES

We used STRUCTURE on these data to estimate the genetic admixture of the African American and Mexican American participants. Based on our results we estimate that African Americans in NHANES III and 1999–2002 surveys have on average 13 and 9% European ancestry, respectively. We report highly divergent estimates of average percent Asian ancestry for Mexican Americans in NHANES III (44) and NHANES 1999–2002 (2). However, based on our FST results of these markers in HapMap 3, this result is likely a result of a lack of Asian AIMs available in the NHANES 1999–2002 population.

We next identified outliers of genetic ancestry compared with self-identified race/ethnicity (**Table 2**; **Supplementary Figures S1**–**S4**). Overall, we found nearly all of the self-reported non-Hispanic whites in NHANES (>97.5%) clustered consistently with the reference samples of European ancestry (>60%; **Table 3**). At a stricter threshold of >90% European ancestry, at least 90% of self-reported non-Hispanic whites were concordant with genetic ancestry in NHANES III and 1999–2002.

**Table 2:** Clustering of NHANES participants with samples representing three continental ancestries from HapMap 3.

	European	African	Asian
**NHANES III**			
Non-Hispanic whites	0.96 (0.11)	0.02 (0.07)	0.03 (0.08)
Non-Hispanic blacks	0.13 (0.16)	0.82 (0.17)	0.04 (0.07)
Mexican Americans	0.44 (0.21)	0.07 (0.07)	0.49 (0.23)
Other	0.49 (0.27)	0.19 (0.21)	0.32 (0.33)
**NHANES 1999–2002**			
Non-Hispanic whites	0.98 (0.03)	0.01 (0.03)	0.01 (0.03)
Non-Hispanic blacks	0.09 (0.15)	0.89 (0.15)	0.02 (0.04)
Mexican Americans	0.98 (0.05)	0.01 (0.03)	0.01 (0.03)


*Average proportion (variance) of membership to continental ancestry is shown for each self-identified racial/ethnic group by NHANES.*

**Table 3:** Concordance between self-reported race/ethnicity and inferred genetic ancestry in NHANES.

	NHANES III	NHANES 1999–2002
**European Americans**		
50% Threshold	98.8 (2590)	>99.9 (3998)
60% Threshold	97.7 (2355)	99.8 (3993)
75% Threshold	93.9 (2460)	99.4 (3979)
90% Threshold	90.0 (2335)	96.8 (3873)
**African Americans**		
50% Threshold	94.5 (1988)	62.2 (1308)
60% Threshold	90.9 (1913)	60.6 (1275)
75% Threshold	73.9 (1554)	55.2 (1161)
90% Threshold	44.0 (927)	41.6 (876)


*Concordance is calculated at various thresholds representing average proportion of membership to continental ancestry (HapMap 3) as determined by STRUCTURE. Sample sizes for each category are given in parentheses.*

Similarly, most non-Hispanic blacks (>60%) clustered with reference samples from West Africa as expected (>60%; **Table 3**). In comparison with the European Americans, we observed less concordance between self-reported race/ethnicity and a single ancestral population among non-Hispanic blacks. At the strictest threshold (90%), only 44% of self-reported non-Hispanic blacks were concordant with African continental ancestry. However, analogous to diminished FSTs between African Americans and European Americans, these results are expected given recent admixture events between the two groups (Winkler et al., 2010).

**FIGURE 2:**
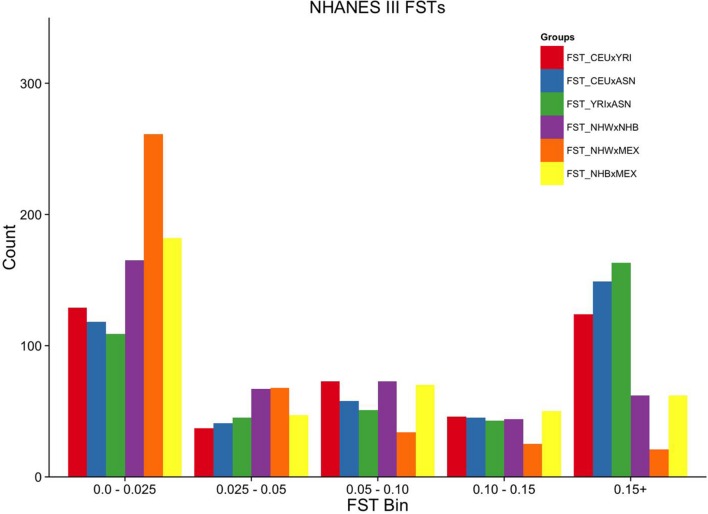
**Ordinal grouping of FSTs for markers genotyped in NHANES III.** Along the *X*-axis are five ordinal FST groupings of increasing differentiation. *Y*-axis is the count of markers. Pairwise comparisons are indicated by the color legend to the right of the figure.

**FIGURE 3:**
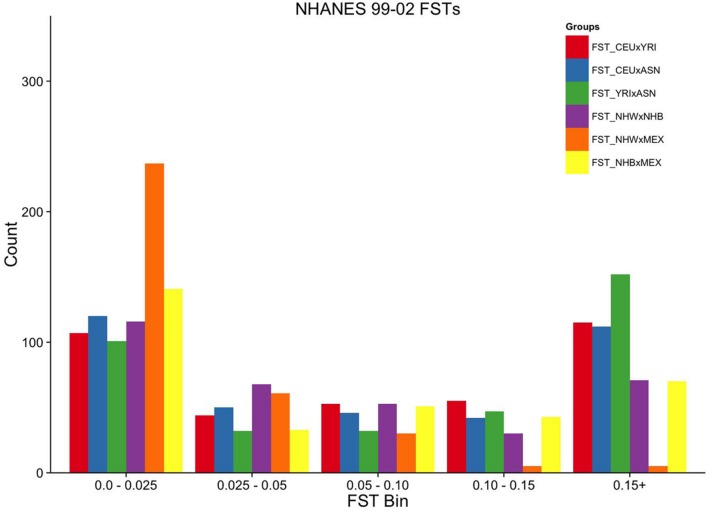
**Ordinal grouping of FSTs for markers genotyped in NHANES 1999–2002.** Along the *X*-axis are five ordinal FST groupings of increasing differentiation. *Y*-axis is the count of markers. Pairwise comparisons are indicated by the color legend to the right of the figure.

Overall, the proportion of the membership of Mexican Americans in the European, African, and Asian clusters was found to be 0.44, 0.07, and 0.49 in NHANES III and 0.98, 0.01, and 0.01 in NHANES 1999–2002, respectively (**Table 2**). Self-reported Mexican Americans from NHANES III clustered more with the Asian continental ancestry cluster than self-reported Mexican Americans from NHANES 1999–2002. This difference may reflect the larger proportion of markers with higher pairwise FSTs between Asian and European groups available in NHANES III (**Figure 2**) compared with NHANES 1999–2002 (**Figure 3**). A comparison of the principal components analysis (PCA) of the Mexican Americans in NHANES III and 1999–2002 reveals that of the three groups, self-reported Mexican Americans are the most similar to the European genetic cluster (**Supplementary Figures S5** and **S6**). However, the Asian admixture is more pronounced in our analysis of the Mexican Americans in NHANES III than NHANES 1999–2002, consistent with our results from the STRUCTURE analysis.

## Discussion

Adjustment for potential population stratification is an expected step in the quality control process for today’s genetic association studies (Turner et al., 2011). This seemingly basic process, however, requires substantial genetic data depending on the resolution desired. While many epidemiologic and clinic-based DNA collections have genome-wide data available for extensive quality control, not all have such data for a variety of reasons including limited budgets to cover large sample sizes and restricted data use agreements. NHANES is one such dataset devoid of these data, prompting our group to devise strategies to conduct genetic association studies sans genome-wide data. Here we show that sufficient proxies for AIMs can be identified from candidate gene and GWAS-identified index variants using basic population genetic metrics (FST). We also demonstrate that for the majority of NHANES (which is non-Hispanic white), self-identified race/ethnicity is highly correlated with broadly defined genetic ancestry. Depending on the population, the strategy outlined here can be applicable to other large datasets that lack genome-wide data.

As has been reported for other large datasets (Dumitrescu et al., 2010; Hall et al., 2014; Banda et al., 2015; Bryc et al., 2015), we find self-reported race/ethnicity is an especially effective proxy for genetic ancestry in NHANES in self-identified non-Hispanic whites, where 90% of self-identified non-Hispanic white participants met a strict threshold for membership (90% clustering with Europeans). Previous studies have suggested that few AIMs are required to estimate global genetic ancestry for European Americans (Kodaman et al., 2013), and it is likely that the limited genetic data available in NHANES are more than sufficient to adjust for population stratification for this group.

As expected, we find self-reported race/ethnicity is less correlated with genetic ancestry in self-identified non-Hispanic blacks and Mexican Americans. Among self-identified non-Hispanic black participants, we report an average 13% European ancestry in the NHANES III and 9% in the NHANES 1999–2002. These values are lower in comparison with other estimates of average European ancestry hovering between 15% (Yaeger et al., 2008) and 25% (Reiner et al., 2005; Banda et al., 2015; Bryc et al., 2015; Dumitrescu et al., 2015). Our estimates, however, fall in the range of another study which sampled across 10 African American populations whose average European admixture varied from 6.8% (Jamaica) to 22.5% (New Orleans; Parra et al., 1998). Our data are also in agreement with recent lower estimates of average European admixture (∼8%) in the Southern Community Cohort Study (Kodaman et al., 2013). Despite the measurable admixture, previous studies have suggested that relatively few AIMS are required to estimate global ancestry in African Americans (Ruiz-Narvaez et al., 2011; Kodaman et al., 2013), suggesting that the genetic data available in NHANES are sufficient for global genetic ancestry estimates for this population.

This study was limited in the assessment of the genetic ancestry for self-reported Mexican Americans in NHANES. It is unclear from our data whether Asian ancestry is higher in the Mexican Americans that were part of the NHANES III survey or if this is solely an artifact of the genetic markers available in this population. In support of the latter possibility, a recent survey of mitochondrial haplogroups demonstrated that the proportion of Native Americans/Asian haplogroups is similar between NHANES III and NHANES 1999–2002 (Mitchell et al., 2014), suggesting little if any difference in genetic ancestry between the two surveys for Mexican American participants. It is unlikely that a complete assessment of Mexican American genetic ancestry in NHANES can be accomplished with the nuclear genetic information we have available. Also, the HapMap 3 samples used here are imperfect proxies for continental ancestry. In particular, several studies have demonstrated that use of East Asian samples fails to the capture indigenous admixture inherent in Latino populations (Bryc et al., 2010; Bryc et al., 2015). It may be that other reference populations such as Mexican Americans from Los Angeles (MXL) available in HapMap 3 (International HapMap 3 Consortium, 2010) and the 1000 Genomes Project (Genomes Project 1000 Genomes Project Consortium, 2015) or Mexicans from the Population Reference Sample (POPRES; Nelson et al., 2008) available in dbGaP (Mailman et al., 2007) are more suitable for comparison with Mexican American participants from NHANES. Furthermore, emerging studies of Mexican American ancestry now utilize broader datasets that include indigenous populations of the Americans and genome-wide data (Moreno-Estrada et al., 2014).

To protect the privacy and confidentiality of the participants, the CDC imposes restrictions on genotyping NHANES samples, therefore limitations regarding the quantity of genetic information encountered in this study are unlikely to be overcome in the near future. CDC also restricts analyses of the combined genotype-phenotype dataset such that all analyses must be performed behind the CDC firewall. To perform analyses of these combined genetic data, investigators must either perform them in-person at the CDC’s Research Data Center (RDC) in Hyattsville, MD or send SAS code to the RDC remotely using the Analytic Data Research by Email (ANDRE) portal (Bush et al., 2013). During the course of this study, we have generated principal components (PCs) for NHANES III and NHANES 1999–2002 samples. Because these genetic data were not coupled with phenotype data, NHANES PCs were generated outside the CDC firewall. Ideally, the PCs generated here or by other investigators with access to genetic NHANES could be merged with the phenotype data behind the CDC firewall to ensure that genetic association studies are minimally adjusted for population stratification.

The present study has several strengths and limitations. Major strengths of NHANES include sample size and the racial/ethnic diversity. However, as already mentioned, NHANES III and 1999–2002 have limited genetic data which greatly limits the resolution of genetic ancestry that can be estimated for each dataset as well as for each individual participant. Only global ancestry could be estimated as opposed to local ancestry, and the resolution of the global estimates are dependent on the availability of AIMs. Furthermore, as already noted, we do not have sufficient AIMS to reliably estimate global ancestry among self-identified Mexican Americans, particularly in NHANES 1999–2002. Also, the genetic data available does not completely overlap between NHANES, making comparisons difficult between surveys.

Recommendations on how to properly estimate genetic ancestry and adjust for population stratification in studies involving Hispanic populations are evolving now that large genetic datasets are available for these populations (Manichaikul et al., 2012; Conomos et al., 2016). It has been widely recognized that the term “Hispanic” is broad and can be used to describe Spanish-speaking individuals from various countries encompassing the Americans, the Caribbean, and, in some cases, Europe (Burchard et al., 2005). This broad ethnicity or population designation fails to describe relevant geographical background or history that informs the degree of expected admixture (Burchard et al., 2005). Even within geographically defined groups such as individuals from the country of Mexico, the proportion of admixture (in this case, from Europeans and Native Americans, and to a lesser extent West Africans) can vary greatly (Moreno-Estrada et al., 2014). These challenges are reflected in the NHANES data; that is, among self-described Mexican Americans, we observed less concordance with HapMap 3 reference population data presumably due to insufficient AIMs data and less-than-suitable reference populations for this group. Furthermore, while the concordance for estimated global genetic ancestry between self-identified race/ethnicity and HapMap 3 reference samples was very high for non-Hispanic whites and, to a lesser extent, non-Hispanic blacks, it is worth noting that other studies have demonstrated measurable population structure with increased resolution of genetic data even for groups residing geographically close to one another (Novembre et al., 2008). The limited genetic data preclude our ability to investigate this further in NHANES.

Despite the major weaknesses of NHANES, we demonstrate that global genetic ancestry can be estimated in the largest self-identified groups and that the majority of self-identified labels (non-Hispanic whites and non-Hispanic blacks) are concordant with these estimates. Overall, NHANES remains useful for specific genotype-phenotype studies, and the data and approaches described here can be applied to control for gross population stratification despite the lack of genome-wide data.

## Author Contributions

The listed authors provided substantial contributions to the conception or design of the work (MO, DC), the acquisition (DC, HD), analysis (RG, KB-G), or interpretation of the data (MO, RG, KB-G) for the work; drafted the work (MO) or revised it critically for important intellectual context (KB-G, RG, DC); gave final approval of the version to be published (MO, KB-G, RG, HD, DC); and agreed to be accountable for all aspects of the work in ensuring that questions related to accuracy or integrity of any part of the work are appropriately investigated and resolved (MO, KB-G, RG, HD, DC).

## Conflict of Interest Statement

The authors declare that the research was conducted in the absence of any commercial or financial relationships that could be construed as a potential conflict of interest.

## Footnotes

1 www.nhgeneticvariant.com

2 http://www.cdc.gov/nchs/nhanes/biospecimens/DNAspecimens.htm
