# Supplementary material for: Population Stratification in the Context of Diverse Epidemiologic Surveys Sans Genome-Wide Data
Source: Front Genet. 2016 May 6;7:76. doi: 10.3389/fgene.2016.00076 (PMC4858524; doi:10.3389/fgene.2016.00076)

**Supplemental Figures**

**Supplemental Figure 1.** Principal components 1 and 2 for NHANES III non-Hispanic white participants and HapMap 3 individuals. Genetic ancestry outliers identified by STRUCTURE (defined here as <90% European ancestry) are circled in this figure. NHANES samples and HapMap 3 YRI, ASN, CEU, samples are colored in blue, red, green, and black, respectively.


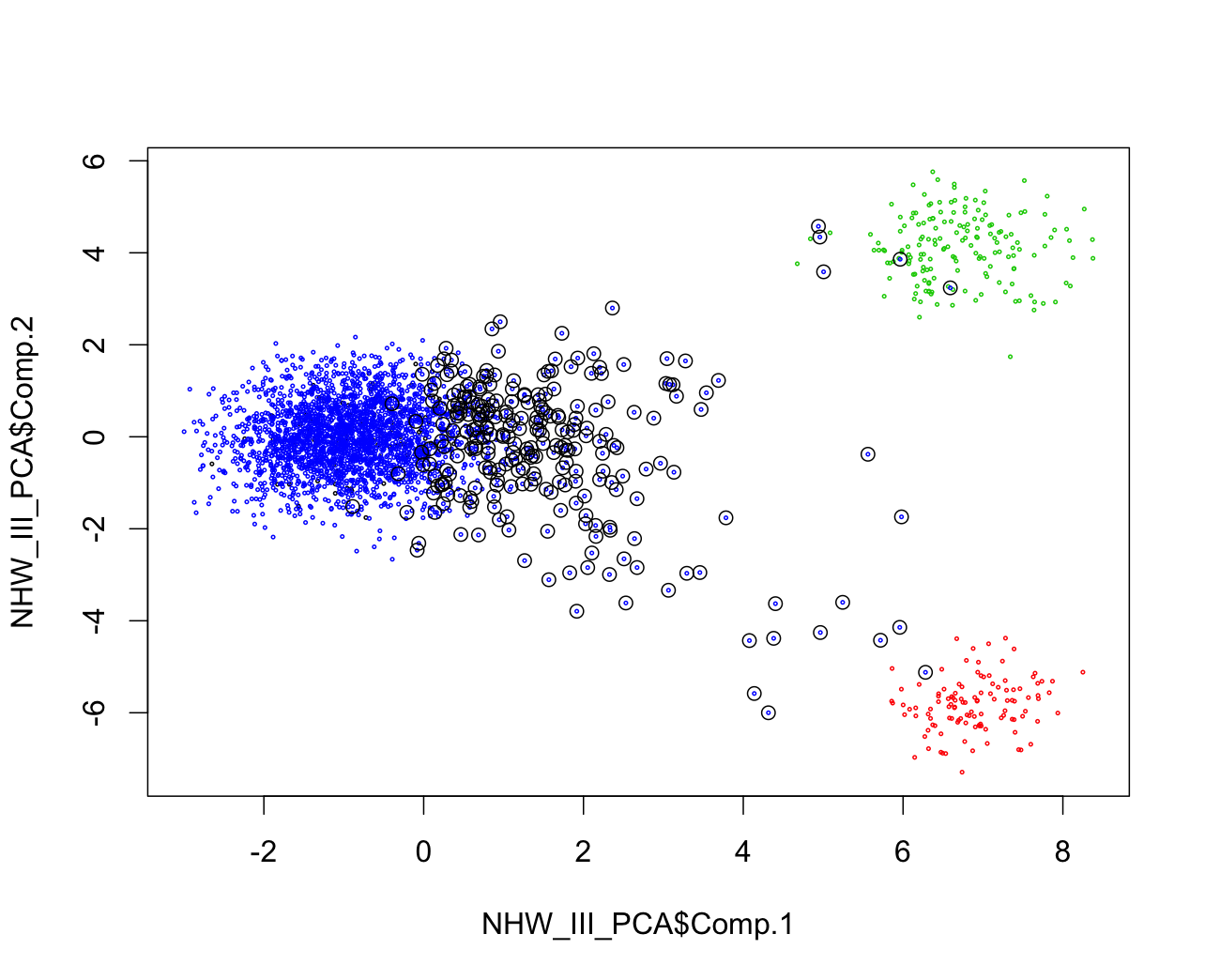


**Supplemental Figure 2.** Principal components 1 and 2 for NHANES III non-Hispanic black participants and HapMap 3 individuals. Genetic ancestry outliers identified by STRUCTURE (defined here as <80% West African ancestry) are circled in this figure. NHANES samples and HapMap 3 YRI, ASN, CEU, samples are colored in blue, red, green, and black, respectively.


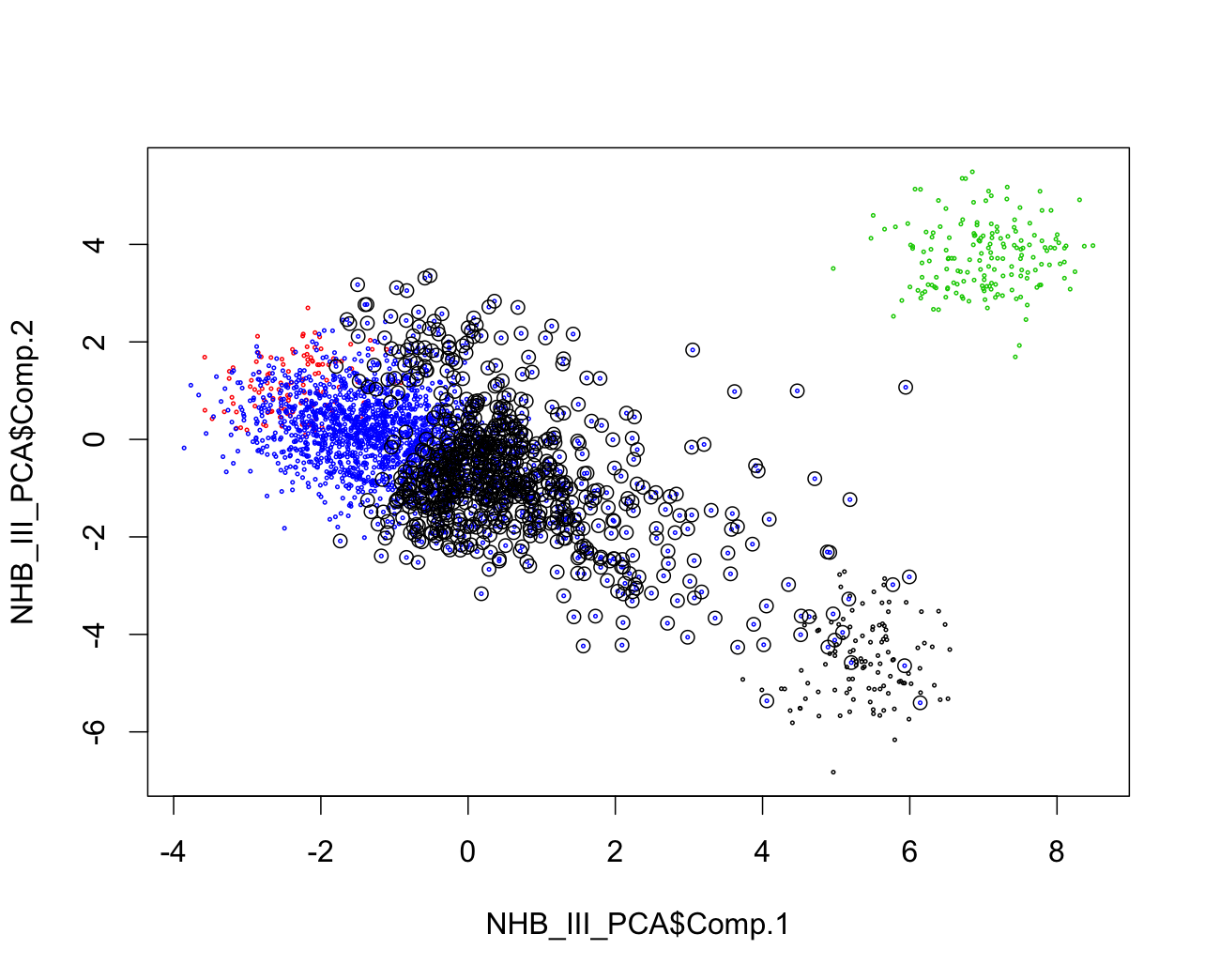


**Supplemental Figure 3.** Principal components 1 and 2 for NHANES 1999-2002 non-Hispanic white participants and HapMap 3 individuals. Genetic ancestry outliers identified by STRUCTURE (defined here as <90% European ancestry) are circled in this figure. NHANES samples and HapMap 3 YRI, ASN, CEU, samples are colored in blue, red, green, and black, respectively.


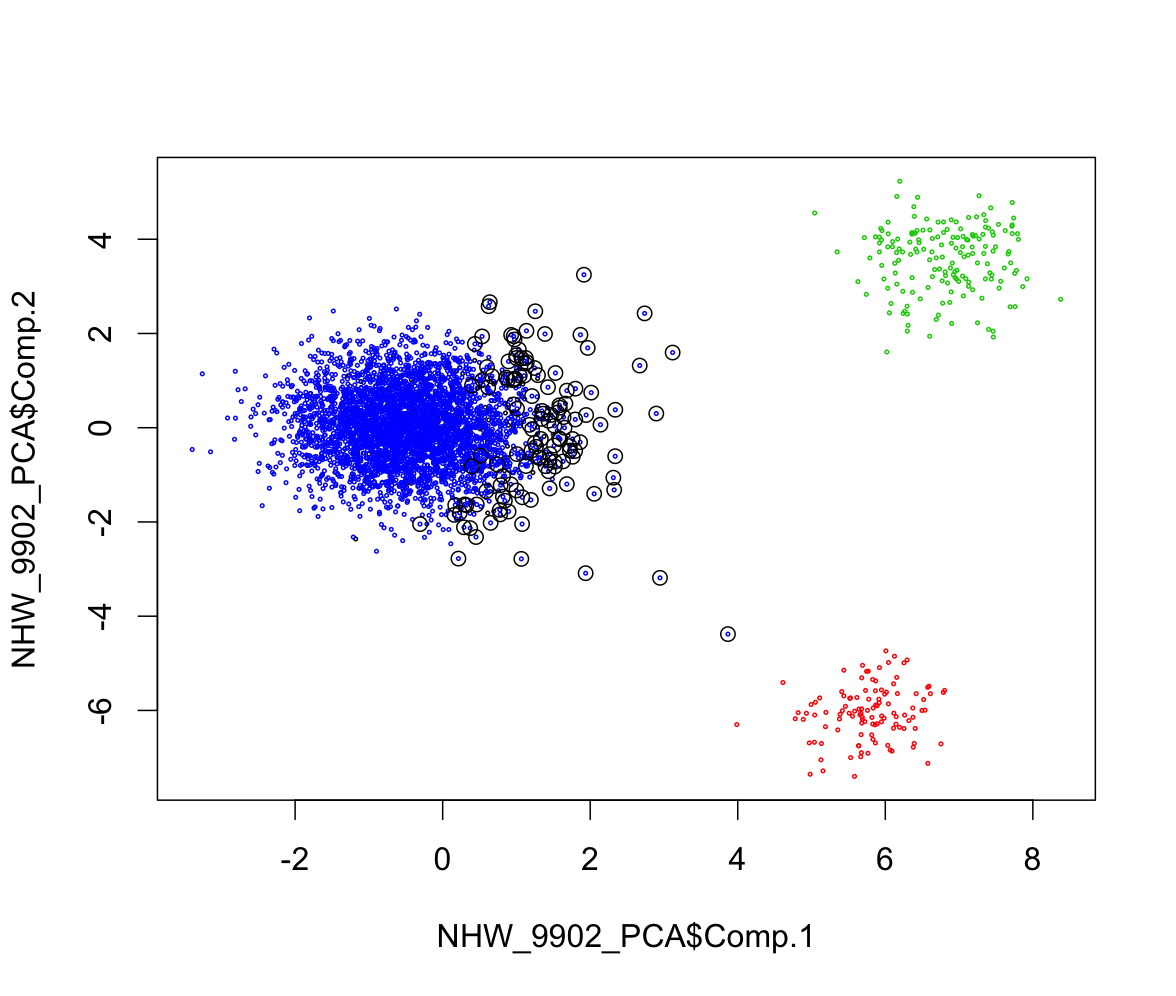


**
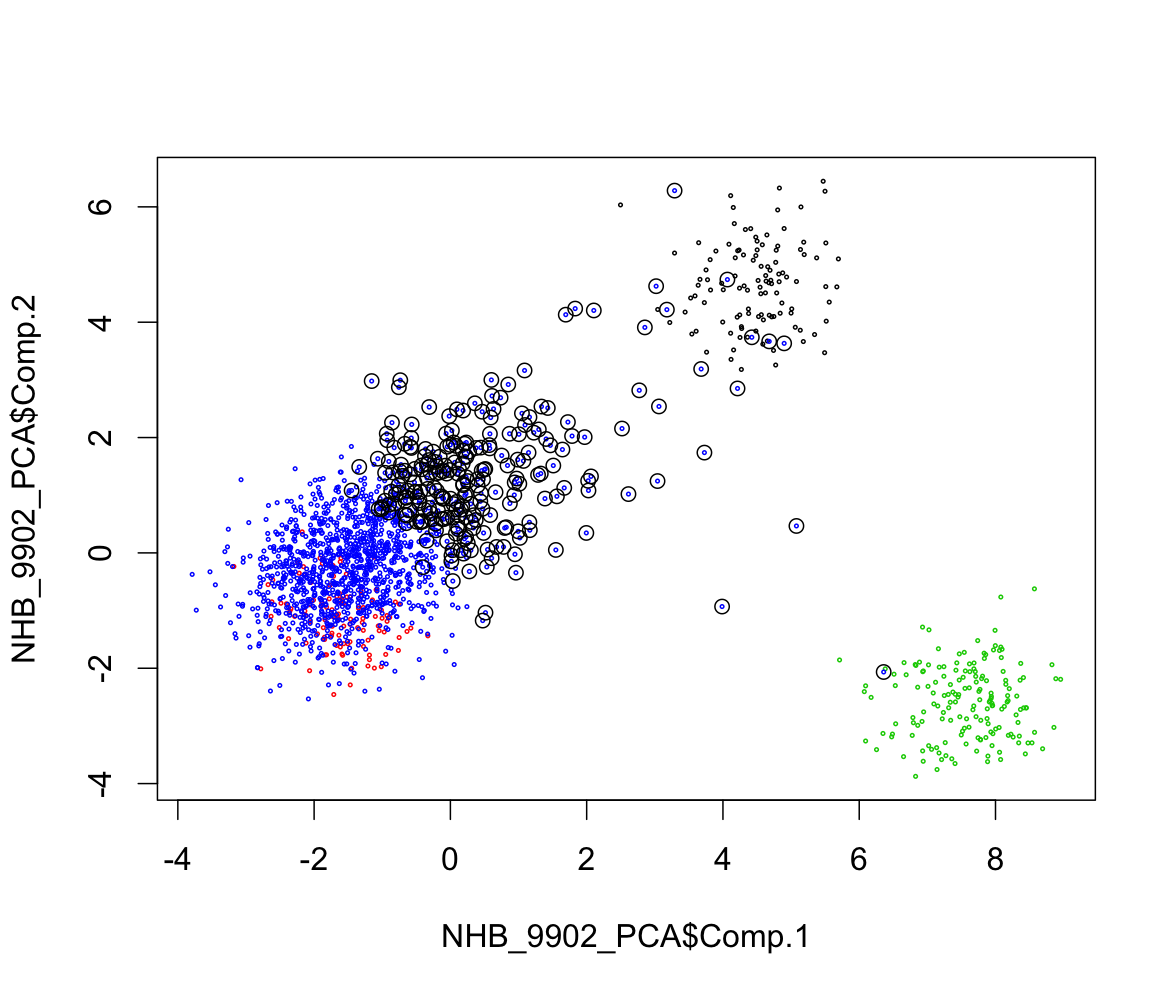
Supplemental Figure 4.** Principal components 1 and 2 for NHANES 1999-2002 non-Hispanic black participants and HapMap 3 individuals. Genetic ancestry outliers identified by STRUCTURE (defined here as <80% West African ancestry) are circled in this figure. NHANES samples and HapMap 3 YRI, ASN, CEU, samples are colored in blue, red, green, and black, respectively.

**Supplemental Figure 5.** Principal components 1 and 2 for NHANES III participants and HapMap 3 individuals. NHANES samples and HapMap 3 YRI, ASN, CEU, samples are colored in purple, blue, green, and red, respectively.


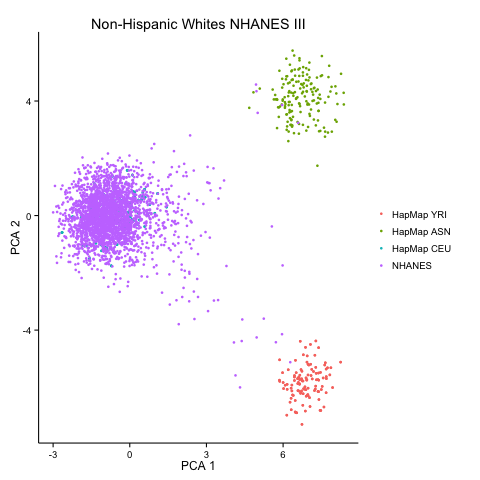


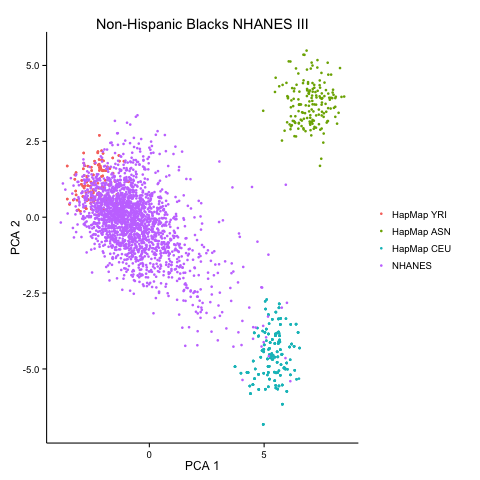


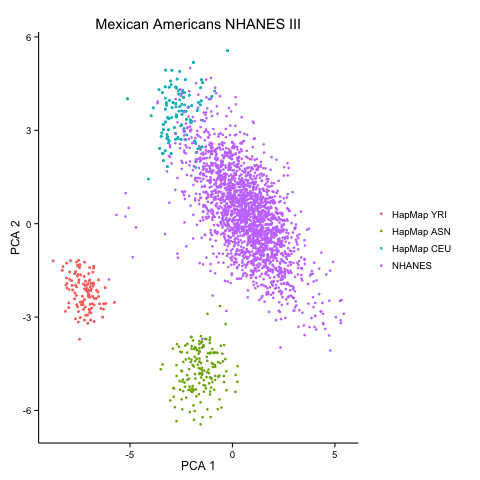


**Supplemental Figure 6.** Principal components 1 and 2 for NHANES 1999-2002 participants and HapMap 3 individuals. NHANES samples and HapMap 3 YRI, ASN, CEU, samples are colored in purple, blue, green, and red, respectively.


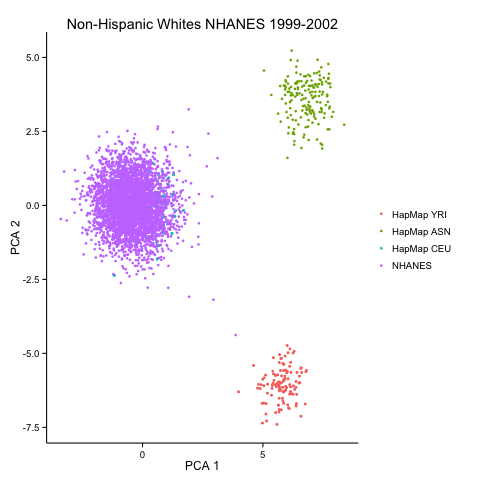


**
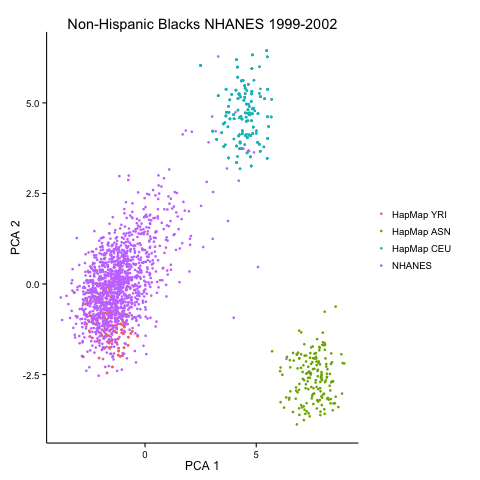
**


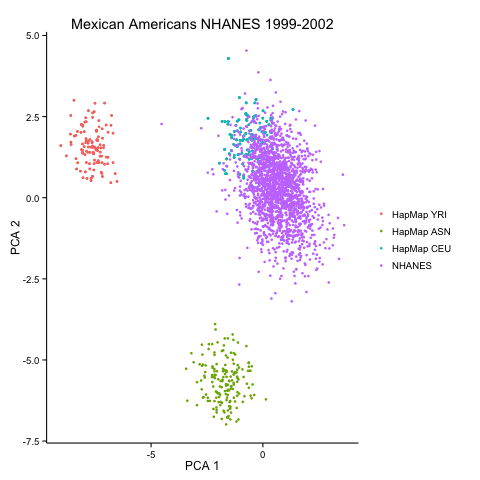

Supplement: Supplementary file 2 [file Data_Sheet_1.DOCX]
